# Combining remote work and training: the experience of participating in the first Virtual EPA Gaining Experience Programme

**DOI:** 10.1192/j.eurpsy.2023.201

**Published:** 2023-07-19

**Authors:** K. Shalak

**Affiliations:** Lviv Regional Clinical Psychiatric Hospital, Lviv, Ukraine

## Abstract

**Abstract:**

Each year the European Psychiatric Association offers early career psychiatrists the opportunity to take part in the Gaining Experience programme, a scholarship that funds short observership placements (between 2 and 8 weeks) overseas in different psychiatric institutions across Europe to ECPs who have completed their psychiatric training. However, since COVID-19 was declared by the WHO as a worldwide emergency on 11th March 2020, it was no longer possible to provide offline overseas observerships. Therefore, the Gaining Experience programme changed its format in 2020-2021 and became virtual. It was in this context, that an Ukranian early career psychiatrist was going to participate in the virtual Gaining Experience programme at the Institute of Psychiatry, Psychology & Neuroscience, King’s College London (United Kingdom) from December 2021. The observership placement had a particular academic focus, the essence of which was investigating psychotherapy training among ECPs in Ukraine, and to examine how it is included in psychiatry training. The sharing of this example of virtual observership shows that it is possible and feasible high-quality and affordable distance learning for ECPs. This training format can be useful not only during quarantine restrictions, but also in conditions of limited travel opportunities.

**Disclosure of Interest:**

None Declared

